# Clinical Findings Leading to the Diagnosis of Sepsis in Neonates Hospitalized in Imam Khomeini and Bu Ali Hospitals, Sari, Iran: 2011-2012

**DOI:** 10.5539/gjhs.v6n4p298

**Published:** 2014-06-24

**Authors:** Roya Farhadi, Mahbobeh Yaghobian, Benyamin Mohseni Saravi

**Affiliations:** 1 Neonatologist MD, Mazandaran University of medical sciences, Sari, Iran; 2 Department of Nursing Management, Mazandaran University of Medical Sciences, Traditional and Complementary Medicine Research Centre, Mazandaran University of Medical Sciences, Sari, Iran; 3 Medical records MSc, Mazandaran University of medical sciences, Sari, Iran

**Keywords:** sepsis, clinical findings, correct diagnosis, medical history

## Abstract

**Background:**

One of the important diseases in neonatal period is sepsis. Clinical sign and symptoms in addition to lab tests are the most important way to accurate diagnosis and prevention of mortality. This study was conducted with the aim of determining the most clinical sign and symptoms which leading to diagnosis of sepsis.

**Materials and Methods:**

This is a descriptive cross-sectional study. The medical records of patients hospitalized in hospitals of Mazandaran University of Medical Sciences during 2011-2012 were reviewed. Variables were age, sex, birth and admission weight, clinical sign and symptoms, methods of delivery, admission and discharge condition, discharge status, the time elapsed between showing the symptom and admission to hospital, gestational age and the result of cultures. The data were recorded in a checklist and analyzed with SPSS and descriptive statistics.

**Results:**

finding showed that 120 patients discharged during period of study with diagnosis of sepsis. Discharged status of 27 (%22/5) were expired. Median age was 1 day with 8 hours SD, length of stay were 12±1 days, gestational age was 34±3 weeks and median birth weight was 2477±977 grams. The median time elapsed between showing the symptom and admission to hospital was 38±31 hours. Blood culture in 10 (%8/3) and urine culture in 8 (%7/6) patients were positive. None of patients have positive lumbar puncture culture. The frequent sign and symptpms in patients were respiratory distress, poor feeding and lethargy.

**Conclusion:**

Early diagnosis of neonatal sepsis is not possible only by specific laboratory exams. Clinical sign and symptoms can help us to prediction and diagnosis of neonatal sepsis. Results of this research revealed that it is not clear which one of manifestations was started first or the second because of medical history sheets don’t show this process.

## 1. Background

Neonatal mortality is a major index in all healthcare systems (Falahi, Joudaki, & Mohseni Bandpey, 2009). Numerous factors have been identified as causes of neonatal mortality. Some Iranian studies report prematurity, respiratory distress, and septicemia as the main causes of newborn death (Naieri, Amini, Oloumi Yazdi, & Dehgan Naieri, 2007; Amid, 2002). Clinical signs and laboratory tests help the correct diagnosis. However, clinical signs are the first thing to attract the attention of the examiner before any laboratory test is made. The frequency and severity of signs are crucial, as they affect the primary diagnosis as well as decisions about admission or treatment protocol. Poor feeding (Hashemi, 2002), respiratory distress, jaundice, apnea and hyporeflexia (Milani, 2007), fever (Fesharakinia, 2004), seizure, cyanosis (Karen & Zaidi, 2010; Kayange, 2010), vomiting (Mosayebi, 2001), skin changes (e.g. petechiae, patchy skin and bruises), tachypnea, apnea, prominent fontanel, abdominal distention (Borna, 2005), respiratory distress (Amid, 2002), and crying (Khalili Matinzadeh, 2007) are among signs reported to be associated with neonatal sepsis. Moreover, focusing on clinical findings and initiating rapid therapy will reduce mortality and complications considerably (Yousefi Mashouf, 2000). Several studies have been conducted so far to assess the effect of disease symptoms on diagnosis. Hashemi (2002) found 500 patients admitted with a diagnosis of sepsis which the most common symptom was poor feeding. Borna (2005) reported hyperthermia and respiratory distress as the most important clinical finding in 97 patients, and poor feeding was found in 86 neonates. Mosayebi (2001) reported hyporeflexia as the most frequent clinical finding, followed by anorexia. Milani (2007) reported 54.8% poor feeding and 29.8% respiratory distress. Fesharakinia observed poor feeding in 80% and temperature alterations (hypothermia or hyperthermia) in 42% of neonates (Fesharakinia, 2004). Kayange reported history of poor feeding, seizure, difficult breathing, retracted breathing, and high and low body temperature (hyper- and hypothermia) as alarm signs for neonatal problems, especially septicemia (Kayange, 2010).

Considering the inconclusive presentations of neonatal sepsis, which may mimic other diseases (Karen & Zaidi, 2010), as well as the importance of timely treatment (Gheibi, 2005), it becomes essential to pay careful attention to clinical findings. Although a definite diagnosis of neonatal sepsis requires blood culture, in cases where there is poor access to highly accurate laboratories or neonatal culture media, one cannot rely on laboratory findings alone and must consider clinical findings, as well. Considering the lack of similar studies in our province, presence of medical students (interns and residents) in Bu Ali and Imam Khomeini Hospitals, and the importance and power of clinical findings in diagnosing sepsis and initiating treatment, admission or referral, we conducted the present study to identify clinical findings which led to a diagnosis of sepsis. The findings of this study may alert students to symptoms, especially during history taking.

## 2. Materials and Methods

This is a descriptive, cross-sectional study conducted in 2011-12 in the newborns ward and the neonatal intensive care unit of Bu Ali and Imam Khomeini teaching hospitals, Sari. The study population consisted of all neonates discharged with a diagnosis of sepsis. Sample size was determined through survey and based on previous studies (Fesharakinia, 2004). The inclusion criteria were cases with a final diagnosis of sepsis (based on the collections of clinical symptoms, laboratory findings, and cultures from blood, urine, cerebrospinal fluid) admitted in 2011-12, and aged 0-28 days. Patients discharged against medical advice before a final diagnosis was made were excluded from the study. We classified the newborn in two categories based on clinical and laboratory data. The first group consisted of newborns that had a positive blood culture in addition to clinical and laboratory findings, yielding a definite diagnosis of sepsis. The second group was comprised of newborns that only showed clinical and/or laboratory findings of sepsis and their blood culture was negative, indicating a diagnosis of suspected sepsis by a neonatologist. The variables studied were age, sex, weight (on birth and on admission), clinical sign and symptoms, method of delivery, general status on admission and discharge, discharge status (resolved, follow-up, expired), the approximate time from onset of symptoms to hospital admission, gestational age, leukocytosis, leukopenia, CRP, and cultures from blood, urine or cerebrospinal fluid. In accordance with the second volume of International Classification of Diseases (ICD-10) as well as pediatric textbooks, birth weight was classified as low (<2500 g), very low (<1500 g), severely low (<1000 g), and macrocosmic (>4000 g) (ISCOD 1992; Martin, Fanaroff & Walsh 2011). The newborn’s gestational age was determined based on the first day of the mother’s last menstrual period, sonographic information from the mother during pregnancy, or Ballard’s score for intrauterine age. Using the gestational age, the neonates were categorized as term (aged 37-42 weeks), preterm (<37 weeks) or post-term (>42 weeks) (ISCOD, 1992; Martin, Fanaroff, & Walsh, 2011). Clinical findings were extracted from the patients’ medical records based on their chief complaint; the laboratory findings recorded in the checklist included leukocyte count, CRP, and cultures from blood, urine and the CSF. In order to identify and correct the shortcomings of the preliminary checklist, a pilot study was conducted on at least 30 medical records. We collected from the archives those medical records pertaining to patients discharged with a diagnosis of neonatal sepsis during the study period, and used the data to complete the checklists. Data were analyzed on SPSS software using descriptive statistics, chi-square test, and cramer’s phi correlation coefficient. For legal and ethical reasons, the patient and the treating physician remained anonymous and the results were reported in accordance with this rule.

## 3. Results

The findings of the study indicated that in 2011-12, a total of 127 neonates were discharged with a diagnosis of sepsis from Imam and Bu Ali hospitals. Among these, 7 had been referred from Imam Hospital to Bu Ali Hospital due to critical conditions, and thus were only studied once, yielding a total of 120 medical records. These included 50 (41.7%) girls. The mean length of stay was 12±1.1 days, with a mean gestational age of 34±3 weeks, and a mean age on admission equal to 1 day±8 hours. The mean birth weight was 2477±977 g and the mean weight on admission was 2487±998 g. 37 (30%) neonates were admitted to the intensive care and the rest to the neonates’ ward. 119 were born in the hospital and one newborn was born in a taxicab. The interval from onset of symptoms to admission ranged from 3 to 216 hours, with an average of 38±31 hours. 29 (24.1%) were delivered through normal vaginal delivery, 88 (73.4%) were the result of C-section, and 3 cases (2.5%) were not identified in this respect. The contingency table of length of stay and patient’s status on discharge revealed that a total of 27 neonates had expired, with 13 of them staying in the hospital for 1-7 days. The findings indicated that 7 (5.8%) neonates had leukocytosis (white blood cell count > 20000) and 9 (7.5%) had leukopenia (white blood cell count < 5000) (Martin, Fanaroff, & Walsh, 2011). CRP was reportedly positive (positive C-reactive protein report or a quantitative report above 10 mg/dL (Martin, Fanaroff, & Walsh, 2011)) in 46 (38.3%) neonates and negative in 74 (61.7%). 10 (8.3%) newborns had positive blood culture and 8 (6.7%) had positive urine culture. As for the cerebrospinal fluid, the CSF culture was negative in 60 (50%) and the rest had either no order to CSF culture, or their spinal tap had failed. Other characteristics of the neonates are presented in Table 1. The patients’ clinical findings were recorded down to 4 chief complaints, presented in Table 2.

**Table 1:** Characteristics of neonates discharged with a diagnosis of sepsis from Imam and Bu Ali Hospitals, Sari in 2011-12

Discharge Status	Frequency	Percent
Follow-up	74	61
Expired	27	22.5
Resolved	20	16.5
Total	120	100
**Birth Weight**	**Frequency**	**Percent**
< 1000 g	14	12/1
1000-1499 g	10	8/5
1500-2499 g	25	21/1
2500-2999 g	27	23/1
> 3000 g	41	35/1
Total	117†	100
**Gestational Age**	**Frequency**	**Percent**
28 weeks or less	13	11.1
29-32 weeks	15	12.8
33-36	30	25.6
37-40	59	50.4
Total	117‡	100
**Neonate’s age**	**Frequency**	**Percent**
< 1 day	46	38.3
1-7 days	30	25
8-29 days	44	36.7
Total	120	100

† Three cases did not have their gestational age mentioned in their medical records.

‡ Three cases not mentioned in medical records.

**Table 2:** Frequency of clinical findings reflected in chief complaints of sepsis diagnoses admitted to Imam and Bu Ali Hospitals, Sari in 2011-2012

Clinical Findings reflected in chief complaints	First	Second	Third	Fourth	Total

	Frequency (percent)	Frequency (percent)	Frequency (percent)	Frequency (percent)	
Respiratory distress	33 (27.5)	7 (10.4)	0	0	40
Poor feeding	5 (4.2)	14 (20.9)	9 (45)	5 (71.4)	33
Fever	30 (25)	1 (1.5)	0	0	31
Lethargy	18 (15)	0	1 (5)	0	19
Hyporeflexia	7 (5.8)	5 (7.5)	5 (25)	1 (14.3)	18
Icterus	5 (4.2)	9 (13.4)	2 (10)	0	16
Tachypnea	3 (2.5)	11 (16.6)	1 (5)	1 (14.3)	16
Cyanosis	9 (7.5)	4 (6)	0	0	13
Vomiting	1 (0.9)	5 (7.5)	1 (5)	1 (14.3)	8
Coughing	4 (3.3)	3 (15)	0	0	7
Hypothermia	2 (1.6)	3 (5.4)	0	0	5
Seizures	0	2 (3)	1 (5)	0	3
Moaning	0	2 (3)	0	0	2
Apnea	2 (1.6)	0	0	0	2
Restlessness on urination	0	1 (1.5)	0	0	1
Petechiae	1 (0.9)	0	0	0	1
Total	120 (100)	67 (100)	20 (100)	7 (100)	-

The chi-square revealed a significant relationship between weight and patient’s status on discharge (p=0.000) with a Cramer’s phi correlation coefficient equal to 63%. In other words, newborns with higher weights were less likely to expire. Moreover, the chi-square test indicated a significant relationship between gestational age and patient’s status on discharge (p=0.000) with a cramer’s phi equal to 54%. In other words, neonates born sooner (under 28 weeks) were three times more likely to expire.

The contingency table of age and the first chief complaint revealed that the most important finding was respiratory distress in 19 (15.8%) neonates aged less than 1 day, fever in 9 (7.5%) neonates aged 1-7 days, and fever in 14 (11.6%) neonates aged 8-30 days. The contingency table of age and the second chief complaint revealed respiratory distress in 5 (7.4%) neonates aged less than 1 day, jaundice in 5 (7.4%) neonates aged 1-7 days, and poor feeding in 11 (16.4%) neonates aged 8-29 days. The contingency table of age and the third chief complaint revealed hyporeflexia and poor feeding each in 1 (5%) neonate aged less than 1 day, poor feeding in 6 (30%) neonates aged 1-7 days, and poor feeding in 2 (10%) neonates aged 8-29 days. The contingency table of age and the fourth chief complaint revealed no complaint in neonates aged less than 1 day, and poor feeding in 4 (57.1%) neonates aged 1-7 days. Table 3 summarizes the infectious agents isolated from urine and blood cultures. The contingency table of positive culture and patient’s status on discharge revealed that only 2 neonates with positive blood culture had expired.

**Table 3:** Infectious agents isolated from blood and urine cultures in cases diagnosed with sepsis admitted to Imam and Bu Ali Hospitals, Sari in 2011-12

Agent	Test	

	Blood Culture	Urine Culture
*Escherichia coli*	4	5
*Staphylococcus Saprophyticus*	1	-
*Klebsiella*	2	1
*Gram posetive cocci*	2	-
*Enterobacter*	2	1
Contamination and multiorganism culture	-	1
Total	10	8

## 4. Discussion

A diagnosis of septicemia as clinical neonatal sepsis syndrome is made extremely difficult by the fact that its clinical picture resembles that of other life-threatening disorders such as necrotizing enterocolitis, hyaline membrane disease, and prenatal asphyxia (Edmond, 2010).

The findings of this study revealed that most of the newborns discharged with a diagnosis of sepsis had a birth weight of 3 Kg or higher, which is inconsistent with the findings of Fesharakinia, Sadiienejad, Gheibi and Milani, but almost consistent with those (Amid, 2002; Milani, 2005; Gheibi, 2005; Sadiienejad, 2003; Fesharakinia, 2004).

The most common bacterial agents isolated from cultures were *E. coli* and Klebsiella: Mosayebi et al also showed *E. coli* as the most common agent while Hashemi & Garebaghi reported *E. coli* and nonfermentative Gram negative bacillus (Hashemi & Garebaghi, 2002; Mosayebi, 2001). (Milani 2007) reported the most common agents to be *Staph. aureus*, *Staph epidermidis*, *E. coli*, Enterobacter, and *Salmonella typhi*. Yousefi Mashouf (2000) reported *Pseudomonas aeruginosa* and *Klebsiella* as the most common agents. Shamsizadeh Hayat Davodi et al. (2008) and Gheibi et al. (2005) reported *coagulase-negative staphylococcus*. Mosayebi (2013) reported Flavobacterium that is an uncommon agent for nosocomial infection, the most common cause of neonatal sepsis in an intensive care unit in Kashan.

In terms of clinical symptoms reflected in patients’ chief complaints, we found respiratory distress, poor feeding, fever, illness and hyporeflexia as the most important findings in admitted patients. Other studies have reported poor feeding and fever (Fesharakinia, 2004), poor feeding and respiratory distress Milani 2005), poor feeding (Hashemi, 2002), hyporeflexia and poor feeding (Mosayebi, 2001), fever and poor feeding (Borna, 2005), poor feeding and icterus (Amid, 2002) and hyporeflexia and poor feeding (Sadiienejad, 2003; Kayange, 2010). It appears that the symptoms or chief complaints do not vary greatly in patients and follow a similar pattern. In a study carried out in Iran (Kashan) the most common clinical presentations were respiratory distress, poor feeding, lethargy, fever and jaundice respectively which the first two findings were similar to our results (Masayebi, 2013). Results of another study in Netherlands revealed that increased respiratory support, capillary refill and grey skin are the most important clinical signs identifying late onset sepsis in preterm neonates (Bekhof, 2013). However, as most cases of blood culture turn out negative, it is important to change the current system (i.e. checklists) of history taking, putting more emphasis on the presence or absence of symptoms and their temporal priority: this will not only yield a more accurate assessment of frequency of symptoms, but will also facilitate a correct judgment on the significance of symptoms on clinical history taking. As suggested by Borna et al. (2005), a clinical scoring system for neonatal sepsis may improve diagnosis. In any case, poor feeding has generally been the most frequent symptoms in previous reports and thus appears to be crucial in a diagnosis of sepsis. The World Health Organization has recently announced use of clinical symptoms for diagnosis of critically ill neonates to have high sensitivity and low specificity in countries with limited facilities, and recommends it as guideline for treatment (Edmond, 2010).

The findings of the study revealed that the mean length of stay was 12 days, ranging from 8-12 days which is consistent with the findings of Borna (2005) who reported 12±5 days length of stay for patients who had a positive blood culture in addition to clinical symptoms. Analysis of patients’ length of stay is a valuable factor for health policy-makers to evaluate care expenses, and it appears that sepsis patients tend to have long stays.

27 (22.5%) neonates had expired; in similar studies, Milani (2007) reported 32 (30%), Fesharakinia (2004) 30 (30%), Falahi (2009) 12 (20%), Naieri (2007) 13.6%, and Gheibi (2005) reported 13 (13%) out of 101 patients assessed. Hashemi evaluated 500 neonates and reported 92 (4.18%) deaths (4). It seems that the mortality rate of sepsis in neonates is similar (about 20%) in all studies. It is essential for policy makers to attempt to lower this rate.

As for gestational age, most neonates were born at 37-40 weeks. This is consistent with Amid (2002), who reported most newborns to be term (88.9%). Also, Naieri (2007) reported septicemia to be a common cause of mortality in term neonates. These comparisons may indicate that infection is a threatening factor for term newborns.

## 5. Conclusion

Given the difficult diagnosis of neonatal sepsis and low rate of positive cultures due to different reasons, clinical findings gain more importance and notice to clinical manifestations can be very helpful in the early diagnosis of neonatal sepsis. Therefore, it is crucial to record and document the events and information pertaining to the neonates. We recommend extensive studies to gain sufficient reliability for early detection of neonatal sepsis, such as a scoring system based on research findings.
